# Three-Dimensional Gel Dosimetry in a Simulated Postmastectomy with Expandable Prosthesis Radiotherapy

**DOI:** 10.3390/gels11050335

**Published:** 2025-04-30

**Authors:** Juliana Fernandes Pavoni, Jessica Caroline Lizar, Leandro Frederiche Borges, Patricia Nicolucci, Yanai Krutman, Oswaldo Baffa

**Affiliations:** 1 Department of Physics, Faculty of Philosophy, Sciences and Letters of Ribeirão Preto, University of São Paulo, Ribeirão Preto 14040-901, SP, Brazil; jfpavoni@ffclrp.usp.br (J.F.P.); jessica_lizar@yahoo.com (J.C.L.); nicol@usp.br (P.N.); 2 Radiotherapy Sector, Clinical Hospital of the Ribeirão Preto Medical School, University of São Paulo, Ribeirão Preto 14048-900, SP, Brazil; lfborges@hcrp.usp.br; 3 Soroka Medical Center, Radiotherapy Department, Institute of Oncology, Beer Sheva 8410501, Israel; yanaik@clalit.org.il

**Keywords:** 3D dosimetry, gel dosimetry, breast cancer, expandable prosthesis, radiotherapy, MAGIC-f gel

## Abstract

Postmastectomy radiation therapy (PMRT) is an adjuvant treatment for breast cancer. Some mastectomized women undergoing PMRT can have breast reconstruction with expander implant reconstruction. However, the expander implant contains a magnetic metal port for its inflation, and in patients with a high risk of recurrence, the PMRT is performed before the expander replacement. The difficulties in radiation treatment near high-Z metals are mainly due to dose alterations around them. Therefore, this study proposes using a realistic breast phantom and gel dosimetry to investigate the effects of the metallic parts of the expandable prosthesis on the 3D delivery of the treatment. A conformal radiation treatment was planned and delivered to the gel phantom with the metal port. MAGIC-f gel was used with magnetic resonance imaging for dose assessment. The treatment plan dose distribution was compared to the measured dose distribution by gamma analysis (3%/3 mm/15% threshold). A significant gamma fail region was found near the metal port, corresponding to a dose reduction of approximately 5%. This underdose is within the tolerance threshold for dose heterogeneity established by the International Commission on Radiation Units (ICRU), but should be considered when treating these patients.

## 1. Introduction

Postmastectomy radiation therapy (PMRT) is an adjuvant treatment recommended for patients with advanced breast cancer or high-risk pathological features. It improves local control and overall survival [1]. Most mastectomized women can have breast reconstruction, which aims to restore the aesthetic appearance of the breast. For women undergoing PMRT who choose implant reconstruction, two-stage surgery with expander-implant reconstruction is the most commonly used approach [2,3,4,5]. Firstly, the tissue expander is inserted during the mastectomy procedure. It is in the form of a silicone bag and is positioned above the patient’s breast muscle, in the prepectoral position. Postoperatively, the expander is inflated with a saline solution until it reaches the desired volume. The inflation process is possible because a magnetic valve is used in the expander, which is located externally by a magnetic locator, allowing the saline solution to be injected in the right spot. After achieving the desired volume, a second surgery replaces the expander with a permanent prosthesis [6].

The radiotherapy timing during the two-stage surgery for breast reconstruction can vary depending on the chemotherapy scheme. For patients with adjuvant chemotherapy, the first surgery occurs, and then the tissue is expanded during the chemotherapy treatment. The permanent prosthesis is implanted after the chemotherapy is completed, and is followed by the PMRT with the irradiation of the permanent prosthesis. On the other hand, for patients who receive neoadjuvant radiotherapy, the first surgery occurs after the chemotherapy is completed, and only after some months of tissue recovery, the permanent prosthesis implantation surgery is performed. This difference occurs because delaying the initiation of PMRT until the implant exchange is not justified for patients with a high risk of recurrence [2]. However, irradiating the expander with the metal port makes radiotherapy challenging due to the high-Z material. Hence, to achieve an accurate radiation therapy treatment, it becomes crucial to consider the dosimetric effect of the metal port and its artifacts on computer tomography (CT), which is a critical element in the treatment plan.

The difficulties in the radiation treatment near the high-Z metal are mainly due to dose alterations around it. Although some studies report negligible dose alteration near the metal port [7,8,9], most highlight considerable dose perturbations depending on beam angle, energy, and geometry [10,11,12,13,14,15,16,17,18,19,20,21,22,23,24]. The accuracy of the treatment planning system (TPS) in modeling the dosimetric effect of the high-Z material is also a limiting essential point since most current TPSs are not calibrated or validated for the high-density metallic port in the tissue expander [11,20,25]. Advanced algorithms, such as collapsed cone convolution, have been recommended to improve calculation accuracy when beams pass through metallic implants [26].

In summary, several experimental and computer-simulated studies about the effects of the metal port in the expandable prosthesis in radiotherapy have different results. All the previous experimental studies are based on dosimetry made in a plane, when film was used, or at small volumes where thermoluminescent dosimeters (TLDs) were employed. These approaches are inherently limited in spatial resolution and fail to capture the full complexity of three-dimensional (3D) dose distributions in heterogeneous media. A genuine three-dimensional dosimetric study of this problem has yet to be performed.

Gel dosimetry is a powerful tool that enables true three-dimensional (3D) dose mapping in radiotherapy, as radiation traversing each small gel volume leaves a measurable imprint. This capability allows for the accurate reconstruction of the total 3D dose distribution delivered during treatment [27,28,29,30,31]. Numerous studies have demonstrated gel dosimetry’s clinical relevance and versatility, particularly in complex radiation scenarios where conventional dosimetric tools are limited [32,33,34,35,36,37,38,39,40,41]. These gels respond to ionizing radiation through physical or chemical changes proportional to the absorbed dose.

Among the various gel systems, polymer gel dosimeters—such as MAGIC (Methacrylic and Ascorbic acid in Gelatin Initiated by Copper)—have shown excellent performance in 3D dose verification for radiotherapy applications [27,28,29,30,31]. These dosimeters operate via radiation-induced polymerization and cross-linking reactions within a gelatin matrix, altering physical properties such as T2 relaxation times, which can be quantitatively assessed using magnetic resonance imaging (MRI) [27,28]. The changes in T2 relaxation are directly proportional to the absorbed dose, enabling the reconstruction of high-resolution 3D dose distributions through MRI.

Molecular oxygen, a strong free-radical polymerization inhibitor, significantly influences the performance of polymer gel dosimeters. Oxygen competes with monomer radicals, effectively quenching the polymerization reaction and reducing dose sensitivity [27,28]. To mitigate this effect, antioxidants such as ascorbic acid are incorporated into the gel formulation. These compounds serve as oxygen scavengers, binding to dissolved oxygen and reducing its inhibitory impact, enhancing the polymerization efficiency even under normoxic conditions.

Furthermore, adding formaldehyde to the MAGIC formulation results in MAGIC-f gels, which exhibit improved thermal stability and dosimetric sensitivity due to enhanced cross-linking within the polymer network [30,35]. This makes MAGIC-f particularly suitable for precise and reliable 3D dosimetry in clinical and research settings involving complex dose distributions and high-gradient radiation fields.

This technique is particularly valuable in scenarios involving tissue inhomogeneities or the presence of metallic implants, where traditional dosimetry methods struggle to deliver accurate results [36,39,40,41]. Studies have validated the use of polymer gel dosimetry in end-to-end tests for intensity-modulated radiation therapy (IMRT), volumetric modulated arc therapy (VMAT), and craniospinal irradiation, highlighting its clinical relevance [32,33,34,35].

Thus, this study proposes the use of polymer gel dosimetry—specifically, MAGIC-f gel combined with MRI readout—to investigate the dosimetric perturbations caused by metallic components in tissue expanders used during PMRT. The goal is to provide more accurate and clinically meaningful data to optimize treatment planning and delivery in this complex clinical setting.

## 2. Results and Discussion

**Figure 1:**
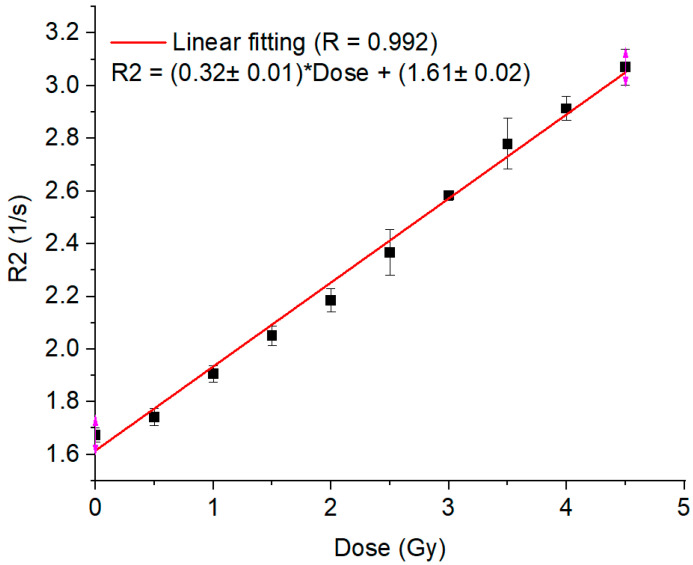
Calibration curve relating R2 to dose. The error bars correspond to the standard deviation of the R2 values inside the analyzed ROI. The pink arrows mark the endpoints of the error bar range.

**Figure 2:**
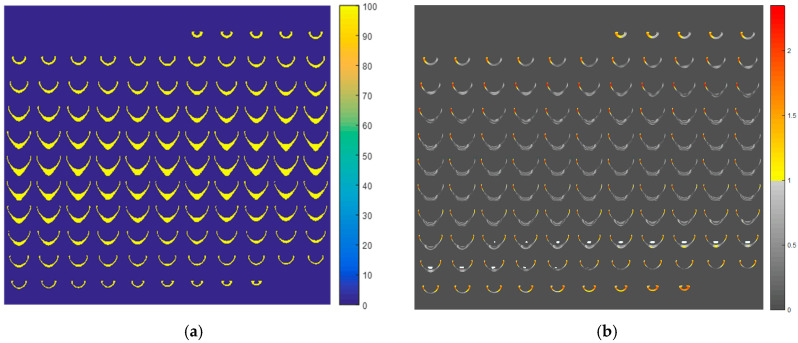
Normalized dose distribution for all slices measured with gel dosimetry (**a**) and gamma analysis (3%/3 mm/10% threshold) comparing the gel results with the TPS expected dose distribution (**b**). Note the metal port positioning representation in the white color inside the gel phantom with the gamma results.

**Figure 3:**
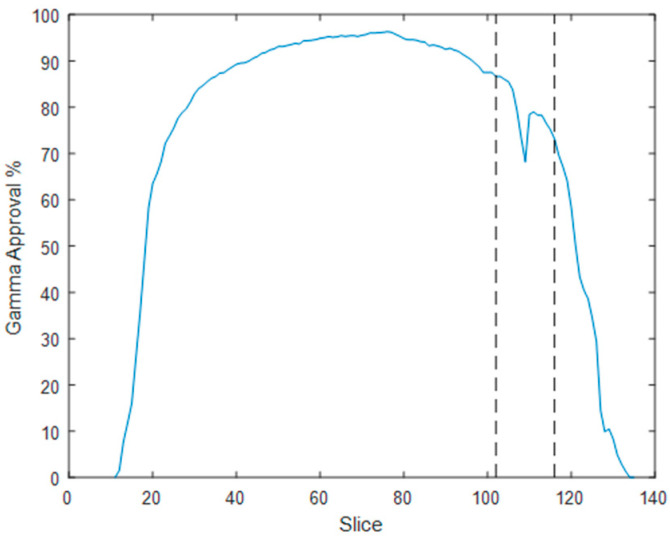
Gamma pass rate for all MRI slices measured. The two vertical bars define the region where the metal port was positioned in the phantom (slices 102 to 116).

A linear relationship between R2 and dose, with a Pearson correlation coefficient of 0.992, was achieved in the gel calibration curve for doses ranging from 0.5 to 4.5 Gy (Figure 1). The mean uncertainty in the gel calibration was 3.0%. This gel batch’s mean dose resolution (or the ability to resolve doses with polymer gel dosimeters) was 0.07 Gy across the entire calibration dose range, decreasing to 0.02 Gy at the 3 Gy dose level [42]. The linear relation in the calibration curve allowed the calculation of the normalized dose distributions for all the MRI slices (Figure 2a). The gel and the TPS dose distribution were compared by gamma maps (3%/3 mm/10% threshold) for all slices (Figure 2b). Most of the gamma failures appear in the thin walls of the phantom, which may be due to oxygen contamination from the phantom walls, inhibiting gel polymerization. Considering the small volume of the phantom, these failures represent an important amount of failure in the measurements, resulting in a lower overall pass rate than the usual acceptance value of 90% or 95% [43]. However, as we aimed to evaluate the influence of the metal port in the irradiation, an evaluation of the gamma passing rates for all slices (Figure 3) showed different behavior. A dip in the gamma passing rate profile of about 10% was detected on the slices with the metal port.

**Figure 4:**
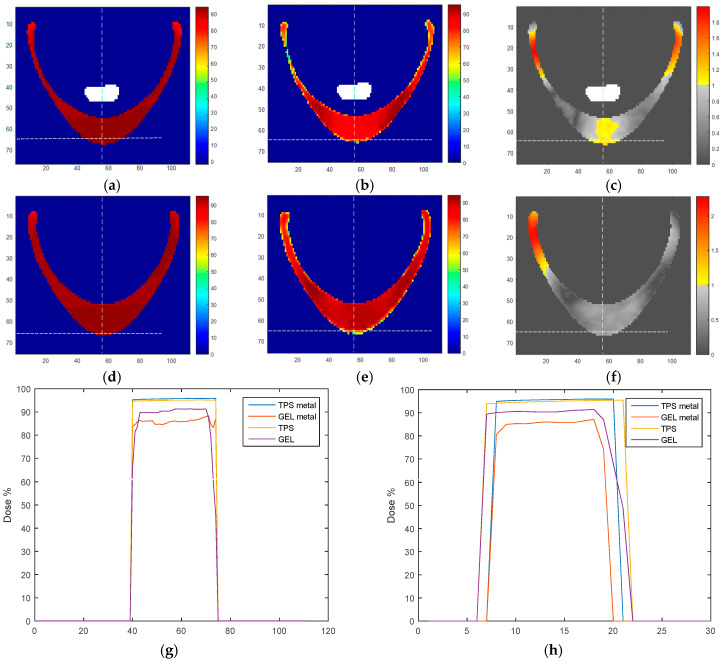
Illustration of the results obtained in the metal region (slice number 109): TPS dose distribution (**a**), gel dosimeter measured dose distribution (**b**), the gamma analysis (3%/3 mm/10% threshold) image (**c**). Note the metal port positioning representation in the white color inside the gel phantom in these Figures. The results obtained in an equivalent slice, without the metal port presence (slice 37), are also presented: TPS dose distribution (**d**), gel dosimeter measured dose distribution (**e**), and the gamma analysis (3%/3 mm/10% threshold) image (**f**). Dotted lines (**a**–**f**) relate to the planning isocenter. Horizontal (**g**) and vertical (**h**) dose profiles are presented with the TPS and the gel dose values; the metal term in the legend indicates the profiles in the presence of the metal.

In a closer examination, the results obtained in the metal region for slice 109, located approximately in the center of the metal port volume, indicate a slight dose difference between TPS (Figure 4a) and the measured dose distribution (Figure 4b). Besides the lateral extremities, where a thin layer of gel is found near the phantom walls, the difference is more pronounced in the central region at the bottom of the figure, resulting in the gamma failures found (Figure 4c). To confirm the relation of the failure region with the metal port presence, we evaluated a slice on the opposite side of the phantom with a similar form, which was not in contact with the metal port. The achieved dose distributions in the TPS (Figure 4d) and gel (Figure 4e) are more similar than those in the presence of the metal. No region with gamma fails was found in the bottom central region of the phantom (Figure 4f). By evaluating the dose profiles in the horizontal (Figure 4g) and vertical (Figure 4h) direction on the lines indicated as dotted lines in Figure 4a–f, we can see a dose reduction in the gel measurements. Still, it is more pronounced in the profile when the metal is present (9.80% ± 1.06% and 9.80 ± 0.43% in the horizontal and vertical dose profiles with the metal versus 4.71% ± 1.40% in the horizontal and 4.21% ± 0.30% in the vertical dose profiles without it). Therefore, it is possible to attribute to the metal presence a mean dose reduction of 5.3%, which is in the shadow of the metal port when irradiated with the tangent fields, and its magnitude is in accordance with the previously published studies [10,12]. A dose variation of this magnitude is important; it is in the threshold of the tolerance limits established by ICRU for the dose heterogeneity accepted in a plan (+7%/−5%) [44]. It should be carefully evaluated during treatment planning to avoid problems in treatment results due to underdosage in this region.

**Figure 5:**
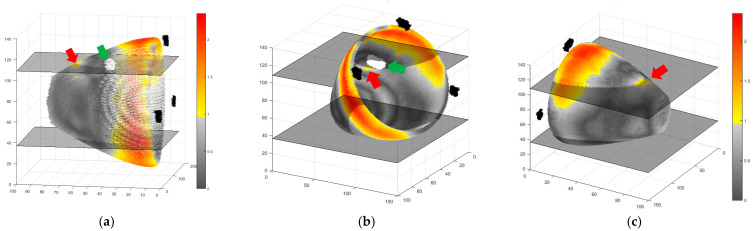
Three-dimensional reconstructions of the gamma results in different views: outside surface (**a**,**c**) and inside surface (**b**). The color bar on the right side of the figure indicates the gamma values, yellow to red points correspond to the failed regions, and the gray points are the approved ones. The three black regions at the bottom of the phantom are the reference marks for image registration, and the white mark in the inner part of the phantom, indicated by the green arrow, corresponds to the metal port position. The red arrow indicates the region of failure due to the metal influence. The positions of the dose distribution slices evaluated are also noted in the figure, one that passes through the metal port and the other on the opposite side of the phantom.

The three-dimensional reconstruction of the gamma results provides another view of the problem and highlights the failures only in the direction of the metal port (Figure 5). In this figure, it is also possible to see the other failures in the thinner region of the breast phantom, which were ignored, as explained before.

Several studies have investigated the influence of metal ports from expandable implants of reconstructive mastectomies on the radiation dose. Thompson et al., using diode detectors, reported dose reductions in the shadow of the port, which are more pronounced for single beam incidences perpendicular to the port axis (30%) than for parallel irradiation with opposed beams (10%) [10]. Damast et al., in a similar ex vivo film dosimetry, found a decreased transmission, particularly with the magnet in the parallel orientation (at 22 mm: 78% transmission with 6 MV, 84% transmission with 15 MV), which added the knowledge of the energy dependence on these deviations [11]. Insignificant increased dose differences according to the treatment technique were also found [19]. Monte Carlo simulation was also employed in this problem. In a realistic patient simulation, using two tangential beams to treat the breast in the presence of the metallic port, the relative reduction compared to dose results without the expander in place ranges from 7% to 13% for the 6 MV beam. It is around 6% for the 18 MV photon beam [12]. In vivo measurements of the patients’ skin dose were also performed, and an average of 7% dose reduction was found in 15 of the 16 evaluated patients. This dose discrepancy was reproducibly located in the ‘shadow’ of the magnet, corresponding to each path of the medial and lateral tangents [17]. Barnes et al., on the other hand, found doses of up to 111.9% of the prescription volume for implant-based reconstruction breasts, reassuring the importance of dose homogeneity to the reconstructive outcomes for patients undergoing PMRT. Park et al. have investigated whether new ESTRO-ACROP target volume delineation guidelines would affect breast complications in radiotherapy in implant-based breast reconstructed patients, finding no major differences [45].

Given the diversity of reported results, investigating the metal port’s influence on the delivered dose remains an open and important issue in the radiotherapy of patients with expandable implants. Hence, the use of different dosimetry approaches to the problem can help shed light on the matter. In particular, gel dosimetry can add innovation to the already used methods, allowing a 3D evaluation of the dose distributions. De Deene and Jirasec have discussed that new technologies and treatment protocols can benefit from 3D gel dosimetry assessment [31]. In this work, gel dosimetry with MAGIC-f has shown that a mean dose reduction of 5.3% is found in the volume in the vicinity of the metal port. Besides being in the range of dose differences found in the already mentioned studies, the results show a spatial perspective of the distribution of the differences between planned and delivered doses. These differences can be even more important in hypofractionated protocols being implemented for breast irradiation in patients with implant-based reconstruction [46].

## 3. Conclusions

A complete 3D dose distribution in a realistic breast tissue phantom was obtained using gel dosimetry to investigate the effects of the metallic components of the expandable prosthesis in the delivery of radiotherapy. A region of gamma analysis failure (3%/3 mm/10% threshold) in the vicinity of the metal port was found, corresponding to a dose reduction of approximately 5%. This is a clinically important underdose and should be considered when delineating a treatment plan for these patients.

## 4. Materials and Methods

In this study, we used glass phantoms to simulate the breast shape and tissue, allowing the metal port positioning as usual in patients. The simulated breast, consisting of the chest wall tissue remaining after the mastectomy surgery, was filled with MAGIC-f gel for a three-dimensional dose distribution measurement. A tangential breast radiotherapy treatment was simulated. The phantom computed tomography (CT) image was obtained without the metal port to avoid the known problems described above. The metal was positioned only afterward, before the phantom irradiation. The measured and planned results were compared by gamma analysis, and the differences found were assumed to reflect the influence of the metal port in this type of treatment.

**Figure 6:**
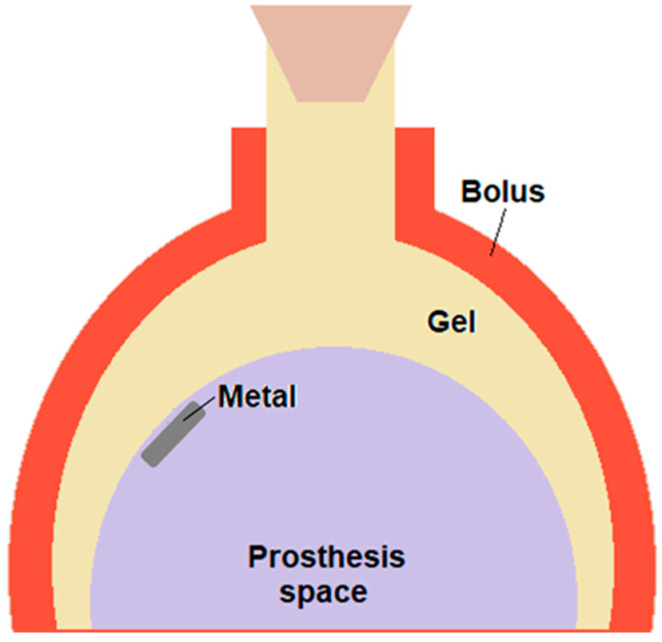
Schematics of the breast-shaped phantom with an illustration of the metal port positioning. A bolus made from dental impression wax was placed above and around the glass bottle for image acquisition and irradiation. It was removed for the MRI acquisition.

Phantom Construction: A borosilicate glass phantom was produced at the local glass workshop according to the breast shape design proposal. A one-liter Erlenmeyer glass flask was modified by having the bottom curved towards the inside to create a volume and simulate the space where the prosthesis is positioned, between the thorax and the breast. A red dental wax layer, approximately 1 cm thick, was applied around the phantom to work as a bolus. The glass phantom had an airtight stopper to prevent air from entering, compromising the gel dosimeter’s performance. The volume available to be filled by the MAGIC-f gel simulating the chest wall tissue was 0.282 L. The metal valve was removed from the SILIMED/470 prosthesis (Silimed Implant Industry, Rio de Janeiro/RJ, Brazil) and was fixed with wax during irradiation at the inside dome of the phantom balloons close to the neck. This valve is a disk with a 17.9 mm diameter and 3.9 mm thick, made of titanium (Ti, density = 4.50 g/cm^3^) and samarium-cobalt magnet (SmCo, d = 8.20–8.50 g/cm^3^). The titanium foil covers the magnet to add extra protection. Figure 6 shows the schematics of the phantom. We also used test tubes with 4 mL volume, 12 mm diameter, and 75 mm length as calibration vials.

**Figure 7:**
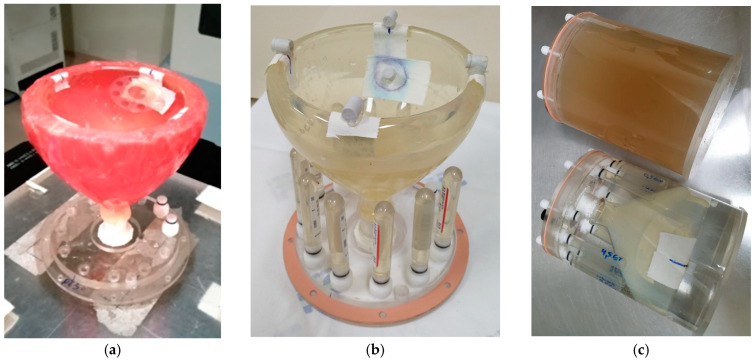
Phantom positioning on the base support for CT image acquisition and irradiation (**a**). Phantom and calibration vials positioning in the base support (**b**), where the markers used for MRI acquisition for image registration and metal localization are also visible. Phantom and calibration vials placed inside the cylindrical vessel for MRI scanning in the lower image, and the cylindrical vessel used to acquire reference MRI in the upper image (**c**). The change in color in the upper flask is due to a large amount of gel. The lower flask has water and a smaller amount of irradiated gel inside the glass balloon.

The breast phantom was positioned in a base stand during image acquisition and irradiation. It was set upside down in this setup, allowing the prosthesis space to be filled with water instead of the prosthesis itself, thereby avoiding air gaps between the prosthesis and the rigid phantom. Reference marks were made to reproduce the phantom positioning during CT acquisition. The metal port was positioned inside the phantom only during the irradiation (Figure 7a). This setup also allowed calibration vials, placed around the phantom, to be scanned together with the phantom (Figure 7b), thus saving scanning time. In this image acquisition, the bolus was removed. Vitamin E marks were used during the MRI acquisition for marking the reference points and the metal port positioning in the phantom in the acquired images. The whole setup was attached to a vessel that fits in the MRI head coil for the MRI acquisition. The remaining internal space was filled with a solution of 99.5% Mili-Q water, 0.3% manganese chloride, and 0.2% sodium chloride in mass percentage. This solution reduces the artifacts in the vial edges generated by the magnetic susceptibility difference between air and vials. A second phantom with the exact dimensions as the cylindrical vessel was constructed to be filled only with gel and used as a reference for the MRI acquisition (Figure 7c, upper image).

Gel Production: Table 1 presents the MAGIC-f gel chemical reagents and their weight mass concentration in the dosimeter. Its preparation has been described previously [22]. Briefly, it starts by dissolving the gelatin in water at room temperature for at least half an hour under continuous magnetic stirring. Then, the solution is heated to 45 °C and kept at this temperature until the complete gelatin melts. Afterward, the temperature is decreased to 35 °C, and the other reagents are added following the order they appear in Table 1, with approximately 2 min between additions. After adding methacrylic acid, the preparation is stirred for an additional 5 min. Finally, the phantoms are filled and left in a refrigerator at 10 °C for at least 12 h before irradiation.

**Figure 8:**
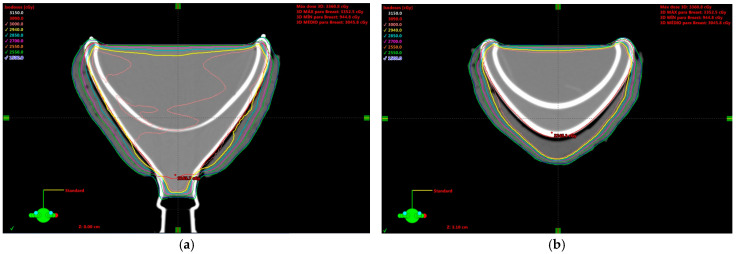
Dose distribution from the treatment planning system for the breast phantom: at the isocenter (**a**) and in the region of the metal port (**b**). The external wax bolus region is visible, as well as the areas inside the glass walls of the phantom and the inner dome.

Treatment Plan and irradiation: The breast-shaped phantoms were taken to the CT-scanner Brilliance Big Bore (Philips Medical Systems, Cleveland, OH, USA) of the Ribeirão Preto Medical School Hospital and Clinics (HCRP) from the University of São Paulo. Reference marks for alignment and isocenter positioning were applied, and radiopaque fiducial marks were positioned on them. The images were acquired following the same clinical protocol used for breast patients. The CT scan was uploaded to the Eclipse (Varian Medical Systems, Palo Alto, CA, USA) treatment planning system (TPS). A treatment plan was designed using two opposed tangential asymmetric beams covering all the phantom (field size of 19 × 20 cm^2^) at gantry angles of 90° and 270° and using 15° wedges with a 6 MV beam. A dose of 3 Gy was calculated and used as it falls within the gel’s linear sensitivity region. Figure 8 shows the dose distribution obtained from the treatment planning system.

**Figure 9:**
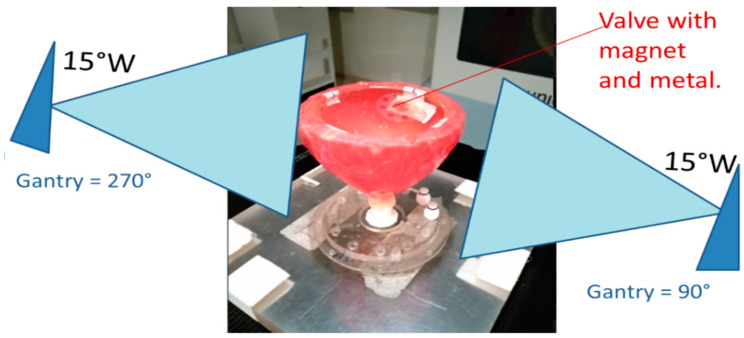
Schematics of the irradiation showing the phantom with the wax bolus and the valve positioned in the inner dome. Note that the inner dome was filled with water during irradiation to simulate the tissue.

The plan was then transferred to the Unique medical linear accelerator using the Aria Record and Verify System from Varian Medical Systems. Before irradiation, the valve with the magnet and metal port was positioned in the phantom, as shown in Figure 9.

The calibration vials were irradiated inside a polymethylmethacrylate (PMMA) plastic block of appropriate thickness to ensure charged particle equilibrium at the radiation energies used.

MRI Acquisition: MRIs were acquired using the Philips Achieva 3T scanner (Phillips Medical Systems, Cleveland, OH, USA). After irradiation, the phantoms were stored in the MRI for one day to complete the polymerization reactions and reach temperature equilibrium before the image acquisition. The images were acquired using a 32-channel quadrature head coil (in which the phantoms were positioned) using a 3D multi-spin echo sequence with eight echo times multiples of 35 ms, repetition time of 1000 ms, and slice thickness of 2 mm. The acquisition matrix was 144 × 144 pixels, and the Field of View (FOV) was 140 × 140 mm^2^. The calibration vials were scanned simultaneously with the breast phantom. The reference phantom was scanned immediately afterward and in the same position in the coil as the first phantom. The reference images were used to correct magnetic field inhomogeneities during image processing. After these corrections, the R2 maps were calculated using a program developed in MatLab^®^ (The MathWorks, Natick, MA, USA). The calibration curve was constructed, relating the R2 value to the respective doses. Given the linear behavior of the calibration curve, all subsequent analyses were conducted on a relative basis. This procedure avoids uncertainties in the dose calibration of vials of different sizes from the phantom [33]. Thus, R2 distributions were normalized by their maximum values, and the same normalization was applied to the TPS dose distribution. The measured and expected dose distributions were compared using a 3D global gamma analysis with a 3% dose difference, a 3 mm distance to agreement, and a 10% threshold.

## Figures and Tables

**Table 1:** MAGIC-f gel components and their respective mass concentrations.

Components	Mass Concentration (%)
Ultra-pure deionized water (Labtools, Ribeirão Preto, SP, Brazil)	82.31
Bovine gelatin—250 bloom (Gelita AG, Cotia, SP, Brazil)	8.33
Ascorbic acid (Sigma-Aldrich, Saint Louis, MO, USA)	0.03
Cooper sulfate (Sigma-Aldrich, Saint Louis, MO, USA)	0.02
Formaldehyde (Sigma-Aldrich, Saint Louis, MO, USA)	3.32
Methacrylic acid (Sigma-Aldrich, Saint Louis, MO, USA)	5.99

## Data Availability

The data that support the findings of this study are available from the corresponding author upon reasonable request.
